# 360-degree projection simulation versus traditional simulation in undergraduate paramedicine education: a pilot randomised controlled trial

**DOI:** 10.29045/14784726.2025.3.9.4.17

**Published:** 2025-03-01

**Authors:** Rachael Vella, Paul Simpson, Liz Thyer

**Affiliations:** Western Sydney University ORCID iD: https://orcid.org/0000-0001-8268-2866; Western Sydney University ORCID iD: https://orcid.org/0000-0003-3875-0061; Western Sydney University ORCID iD: https://orcid.org/0000-0002-8144-4441

**Keywords:** education, extended reality, immersive, paramedics, simulation

## Abstract

**Introduction:**

Simulation is considered a mainstay for teaching and assessment in various clinical fields, including paramedicine. Simulation fidelity in educational practice is constantly changing to accommodate the integration of extended realities (XRs), such as augmented, mixed and virtual realities. However, little research has been undertaken to directly compare these newer, technology-enhanced methods, such as 360-degree projection simulation, with the traditional methods used in undergraduate education. The purpose of this research was to provide a direct comparison, exploring their effect on ratings of self-perceived performance in second-year paramedicine students.

**Methods:**

Using a single-site, parallel randomised controlled, non-blinded trial, participants were randomly allocated to a three-day intensive 360-degree projection (intervention) or traditional (control) simulation programme. Ratings of self-perceived performance were collected using the Seattle University Simulation Evaluation tool at three different time points: after participants’ first simulation as ‘lead paramedic’ on Day 1 of the intensive programme (Rating 1), after their final simulation on Day 3 of the intensive programme (Rating 2) and after their final simulation in a follow-up programme after a nine-week washout period (Rating 3).

**Results:**

Out of the 37 participants randomly allocated, 20 fulfilled the study requirements, with 11 in the 360-degree projection group and nine in the traditional simulation group. Participants consistently reported higher ratings of self-perceived performance in the traditional simulation group, in comparison to the 360-degree projection simulation group (p = 0.04). While no difference was seen between groups after the intensive programme (Rating 2), a notable difference was observed between groups at Rating 3 in favour of the traditional simulation group (p = 0.02).

**Conclusion:**

This pilot study suggested that measures of self-perceived performance were lower when using 360-degree projection simulation spaces. While there may be some benefit to this form of simulation as an adjunct to current traditional methods used, further research, including studies that are appropriately powered and include objective outcome measures, is needed to understand the measure of effectiveness in a practical setting and to inform future educational interventions.

## Introduction

Health simulation provides an engaging, artificial setting for students or practitioners to learn and advance their skills in a realistic environment without posing undue risks to patient or practitioner safety (Pilcher et al., 2012). Simulation places the individual at the focus of their learning, underpinned by Kolb’s experiential learning theory, which suggests that learning stems from experience, both real-world and simulated (Aebersold, 2018; Saleem & Khan, 2023).

Traditional methods of high-fidelity simulation in paramedicine include furnished spaces that mimic the environments in which paramedics practice (Boyle et al., 2007). More recently, extended realities (XRs), such as augmented, mixed and virtual realities (AR, MR and VR, respectively), have been incorporated to create more adaptable, high-fidelity environments (Calvert & Abadia, 2020; Zweifach & Triola, 2019).

Fidelity refers to the degree to which a simulation replicates real-world events (Lioce et al., 2024). High-fidelity simulation varies based on the combination of environment and equipment, where simulators such as task trainers, simulated patients or manikins can contribute to environments. However, the definition of fidelity differs in the instance of XR, which is characterised by the level of reality and virtuality that exists in the environment. If there is a predominant level of real-world stimuli, such as augmented reality, this is considered low fidelity, in comparison to an environment with a predominant level of digital elements, such as virtual reality, which is considered high-fidelity (Le Noury et al., 2022; Tremosa, 2022).

Emerging evidence suggests that simulations incorporating XR offer substantial benefits in paramedic education (Lochmannova et al., 2022; Rees et al., 2020). The environments that can be achieved through technology-enhanced simulation provide replicable experiences that were previously unimaginable (Rees et al., 2020). Increased levels of immersion in technology-enhanced simulation have also been linked to students reporting an increase in satisfaction, confidence and situational awareness in the context of VR (Conradi et al., 2009), 270-degree projection rooms (Barr & Foster, 2017) and 360-degree projection rooms (Williams et al., 2022).

While technology-enhanced methods of simulation have featured more prominently in the paramedicine literature, a comparison with traditional methods in paramedicine and the wider healthcare setting is lacking (Birtill et al., 2021). To date, there has been no study assessing a direct comparison between traditional and technology-enhanced methods of simulation with a common measurement outcome.

Subjective and objectives measures of competence, satisfaction, performance and confidence have previously been used to measure the effectiveness of simulation (Graves et al., 2017; Kiernan, 2018). Most research findings report a negative correlation between the two measures (Carter et al., 2016; Graves et al., 2017; Katowa-Mukwato & Banda, 2016), with a preference for objective outcome measures due to the belief that subjective assessment introduces bias (Koornneef et al., 2018). More recently, arguments have been made against this, with recognition that objective assessment is guided by the attitudes, values and prior experience of the assessor, which may also introduce bias and alter performance ratings (Alastalo et al., 2022; Virk et al., 2020). Multi-faceted assessments, including subjective assessment of the student, need to be considered, as placing students at the centre of their learning results in improved outcomes (Pekrun, 2020; Virk et al., 2020).

In paramedicine, research relating to self-perceived competence is sparse, with a focus on objective, externally rated measures of competence (Hagiwara et al., 2014; Smith et al., 2020). However, nursing emphasises the importance of self-reported measures in student evaluation, evidenced by the development of validated tools that assess self-perceived performance (Mikasa et al., 2013).

The primary aim of this research was to determine if one form of simulation, either traditional or technology-enhanced, would provide greater ratings of self-perceived performance in second-year paramedicine students. The secondary aims were to establish the baseline levels of self-perceived performance using either method of simulation and to determine if these could be maintained after nine weeks.

## Methods

### Reporting

This study is reported in adherence to the Consolidated Standard for Reporting Trials (CONSORT) guidelines (CONSORT, 2010).

### Study design

This pilot study employed a single-site, parallel randomised controlled trial.

### Participants

Participants were second-year students enrolled in an undergraduate paramedicine degree at an Australian university, who had completed 18 months of on-campus study. This involved online and face-to-face theory and practical classes that used traditional simulation and, to a lesser degree, the 360-degree projection environment. Second-year paramedicine students were the most appropriate choice of participant, as they had previously taken part in practical classes involving simulation and had diverse knowledge. In addition they were not in their final semester of study, which would have hindered participation in this research due to conflicts with workplace-based learning requirements. As part of workplace-based learning, the participants had previously completed a three-day non-emergency patient transport placement and a three-week emergency clinical work placement with a jurisdictional ambulance service. Recruitment took place after approval was received from the University Human Research Ethics Committee (H14552). All eligible students were equally provided with the opportunity to participate in the research. Psychological safety was ensured during recruitment, data collection and analysis in line with the approved ethics.

### Study process and data collection

**Figure 1:**
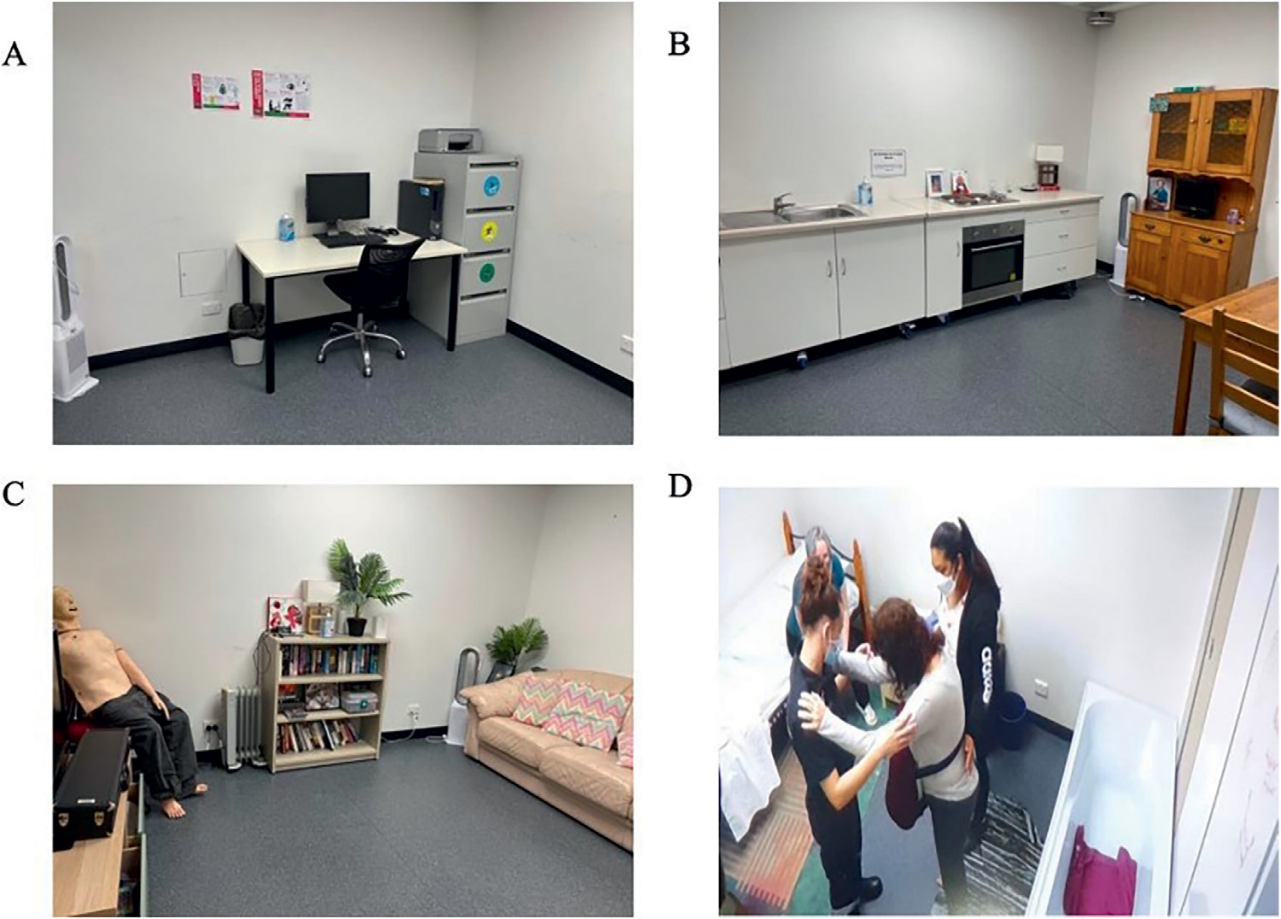
Figure 1. Traditional simulation spaces showing furnished, internal environments, with the patient manikin and the facilitator and students present in the space.

Data collection occurred in a purpose-built paramedicine simulation facility. In the context of this research, traditional methods of simulation were defined as the standard practice at an Australian university, involving static, furnished spaces, designed to represent the internal and external environments in which paramedics may practise (Figures 1A–1C). All internal spaces had additional props to increase fidelity, but no additional audio or visual input. In line with standard practice at the university, the facilitator was present in the simulation space, along with a Laerdal SimMan manikin. The facilitator answered participant questions on behalf of the manikin (Figure 1D).

All facilitators had previous experience in conducting simulations and were provided with detailed patient scenarios prior to each simulation. A face-to-face pre-briefing was held before each session to ensure consistency in the delivery of the scenario and debrief. The debrief was facilitated using the SHARP tool – **s**et learning objectives, **h**ow did it go, **a**ddress concerns, **r**eview learning points, **p**lan ahead – which were familiar to the facilitators and participants (Imperial College London, 2014). This format encouraged debriefing, rather than direct feedback from the facilitator.

**Figure 2:**
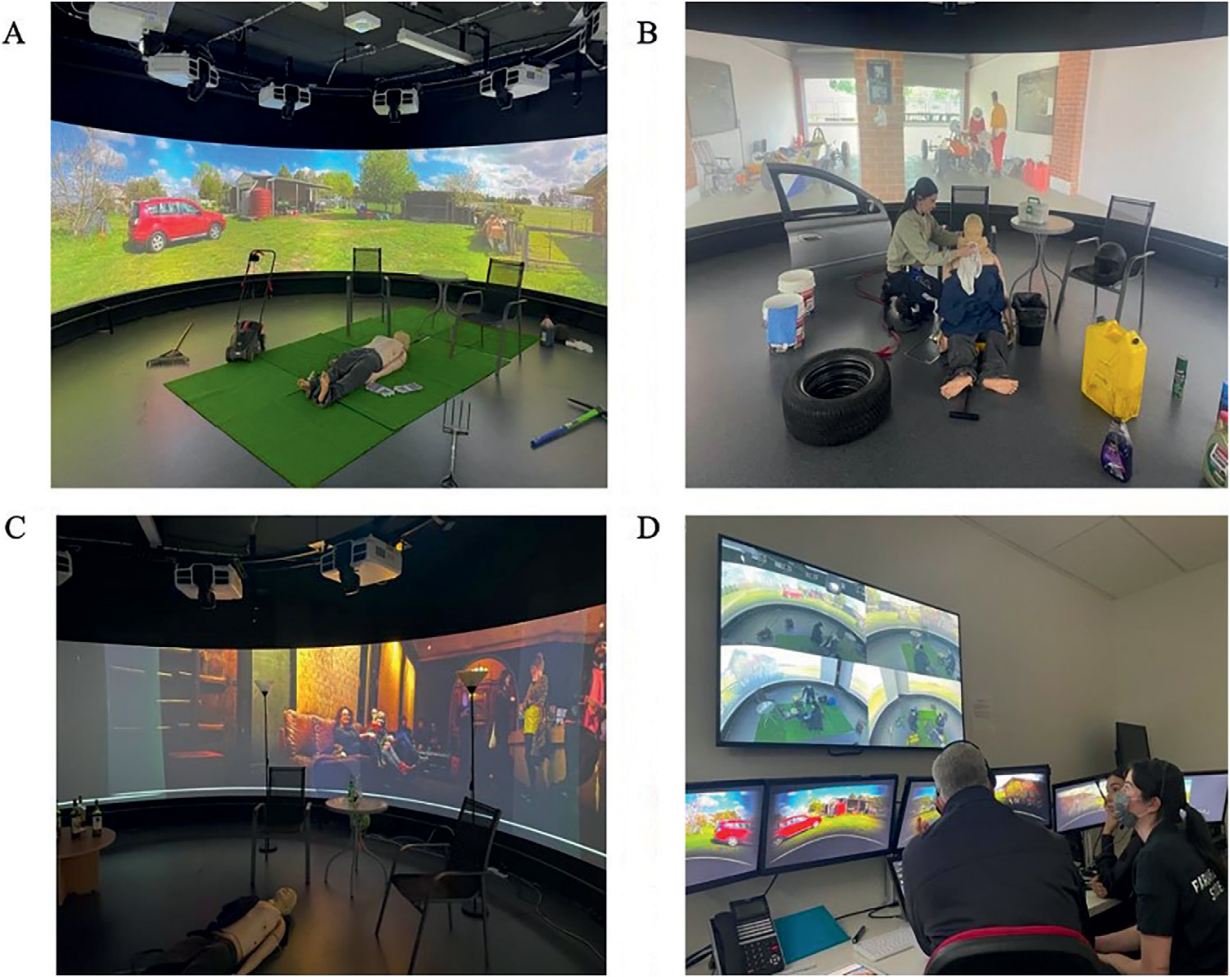
Figure 2. The 360-degree projection simulation space, showing examples of simulated environments and the control room.

The technology-enhanced methods of simulation involved a 360-degree projection simulation space. This method used the projection of video or images onto the walls of the circular-shaped room, accompanied by an audio soundtrack and additional props to increase fidelity. Simulation backgrounds and environments included more complex backgrounds, such as a car garage, jazz bar, farm setting and shopping centre (Figure 2A–2C). The simulation facilitator was not present in the space, instead facilitating from an adjacent control room (Figure 2D), with responses from the manikin being heard through external speakers.

Participation involved a ‘simulation experience’, which entailed involvement as the lead paramedic in both a scenario and an individual debriefing that allowed for reflection on student performance. A group debriefing was undertaken for all participants after the simulation experience. Simulation cases were based on previously taught content, to limit any bias or associated confounding factors, and were peer-reviewed for standardisation. All participants rotated through a range of roles, depending on the design of the simulation. These roles included that of the treating paramedic, support paramedic, bystander or external reviewer; data was only collected from the treating paramedic. The similarities and differences between the simulation experiences of the 360-degree projection and the traditional simulation groups are highlighted in Table 1.

**Table 1.** Similarities and differences between 360-degree projection and the traditional simulation environment.

Simulation features	360-degree projection	Traditional
360-degree projection simulation room	✓	
Furnished simulation rooms		✓
Manikins	✓	✓
Props	✓	✓
Audio soundtrack	✓	
Assessor located within simulation room		✓
Assessor located outside simulation room	✓	
Communication via headpieces	✓	
Standardised scenarios	✓	✓
Debrief using SHARP tool	✓	✓

Based on previous simulation literature (Blackmore et al., 2014; Carter et al., 2016; Dante et al., 2022), each participant was involved in a three-day intensive simulation programme, totalling a 12-hour programme of 15 simulation experiences. Both groups completed the same simulations: 20 minutes in total, followed by a 10-minute debrief. After the completion of the three-day intensive programme, participants continued the normal schedule of their practical and theoretical classes as a part of a nine-week washout period. The concept of a washout period, originally used in clinical trials, refers to a phase during which participants receive no treatment or intervention. This helps to avoid potential biases of misinterpretation of results influenced by prior treatments (Harvey et al., 2021). This time frame was selected due to evidence suggesting that repetitive simulation interventions should occur for a minimum of seven to nine weeks to achieve optimal outcomes, such as an increase in perceived confidence and competence (Al Gharibi & Arulappan, 2020; Mould et al., 2011). After nine weeks, participants returned to complete a half-day programme that encompassed five simulation experiences. The simulation experiences involved new simulations. Participants were placed in the same teams for both interactions, to ensure consistency between the initial and follow-up programmes.

### Randomisation

Participants were enrolled in the study and, through block randomisation, were allocated to either the 360-degree projection or traditional simulation groups. A modified approach to allocation concealment was taken for pragmatic reasons; randomisation was completed before the three-day intensive programme for scheduling and planning but without any knowledge of, or interaction with, the participants before the study commenced.

### Study outcomes

The primary outcome was self-perceived performance, captured using the Seattle University Simulation Evaluation Tool (Mikasa et al., 2013) and used with the author’s permission.

The tool assessed participant self-evaluation in the four domains of assessment/intervention/evaluation, critical thinking / decision making, direct patient care and communication/collaboration. The tool can also assess professional behaviours through external observation; however, this was excluded due to a lack of relevance to the domains of self-perceived performance. Each domain was rated on a Likert scale of 0–5, indicating a range from ‘below expectations’ to ‘exceeds expectations’, resulting in a numerical score out of 20.

Ratings were collected from the treating paramedic in each scenario at three time points: after the first simulation of the three-day programme, to establish a baseline of self-perceived performance (Rating 1); after the final simulation of the three-day programme (Rating 2); and after their final simulation in the follow-up programme that followed the nine-week washout period (Rating 3). The individual completed their rating immediately after the completion of their simulation – in line with the requirements of the tool – where they were offered a five-minute period to independently reflect on the scenario and rate themselves accordingly. Other participants in the scenario did not complete the ratings, as many of the questions were not answerable by their roles in the simulation.

### Sample size

The initial sample size for this research was based on Hagiwara et al. (2014), who compared objective ratings of competence in ambulance nurses pre- and post-simulation exposure. Based on the mean and standard deviation of the ratings before and after simulation, along with a power of 90%, and a 5% two-sided alpha significance level, a sample size of 174 participants, with 87 in each group, was calculated, including 10% to account for loss to follow-up.

The size of the second-year cohort (n = 118) made this impossible. Therefore, a decision was made to set an a priori sample size of 32 participants, with 16 in each group, to inform an initial pilot study.

This research initially met the 32 participants. However, due to state-based restrictions in response to the COVID-19 pandemic – such as the university closures and mandatory two-week isolation for flu-like symptoms – there was an increased dropout rate. Nevertheless, this research still aligned with recommendations of pilot randomised control trials, having a minimum sample size of 20 participants and informing other researchers about the methodological and practical challenges of a full-scale trial (Keys et al., 2021).

### Statistical analysis

Descriptive statistics were generated using SPSS (version 28) to describe participants’ characteristics and assess the normality of data, reported as means with standard deviation. The difference between groups was compared using an independent t-test for numerical data such as age, and a Pearson’s chi-square test for independence for categorical data such as sex and previous degree. When comparing the differences between pre- and post-Likert ratings of self-perceived performance, the data was normally distributed, and an analysis of variance was used to compare within and between subject differences. Statistical significance was established at a probability of 0.05.

## Results

**Figure 3:**
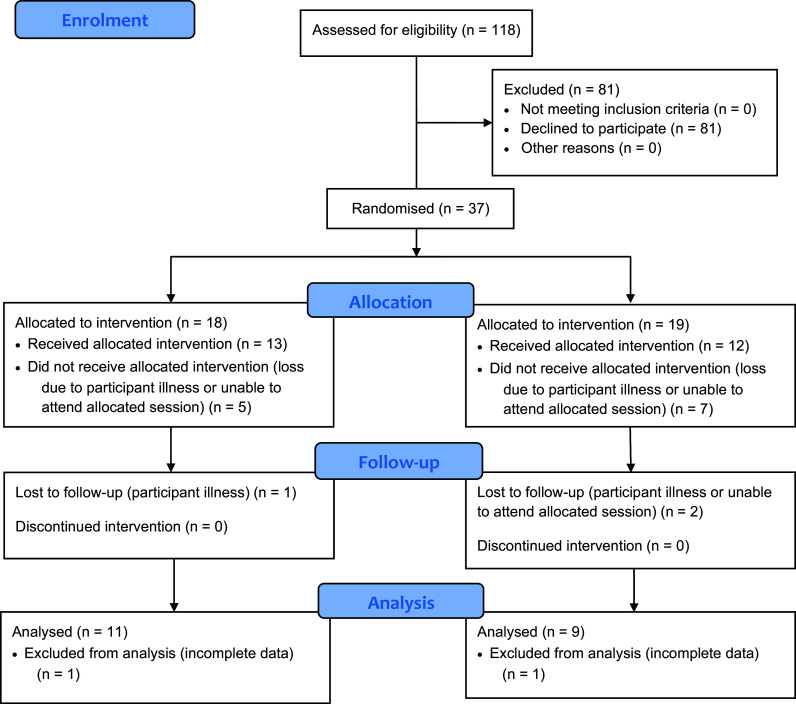
Figure 3. Participant enrolment, allocation and outcomes for analysis.

There were 118 eligible second-year paramedicine students, with a final sample for randomisation of 37 participants. Due to reasons such as sickness or COVID-19 confinement, 17 participants were lost, leaving 20 participants for analysis: 11 in the projection group and nine in the traditional group (Figure 3). Data collection occurred between July and September 2022.

Participant demographics relating to age, sex and previous degree were collected to establish baseline characteristics of the participants allocated to the 360-degree projection and traditional simulation groups (Table 2). No student had completed a degree that would provide them with previous simulation exposure. Most of the participants identified as female, accounting for 65% of the sample size, reflective of the student population. While the age of participants showed a difference between groups, this was not accounted for in the analysis, as age was unlikely to influence the performance of the intervention in comparison to the control in this sample size.

**Table 2.** Characteristics of participants.

Characteristics	Control group (n = 9)	Intervention group (n = 11)	Total (n = 20)	Comparison of baseline statistics (p <0.05)
**Age – years (mean/SD)**	19.89 (1.36)	23.18 (3.49)	21.70 (3.16)	0.02^*^
**Sex – female (number/%)**	4 (44%)	9 (82%)	13 (65%)	0.81
**Previous degree – yes (number/%)**	1 (11%)	5 (45%)	6 (30%)	0.10

n = number of participants; p = probability; SD = standard deviation; * = statistically significant.

After correcting for differences at Rating 1, the traditional simulation group consistently reported higher mean (Xİ) ratings of self-perceived performance in comparison to the 360-degree projection simulation group (p = 0.04) (Table 3). This was different between groups in Rating 3 (p = 0.02), which was after the nine-week washout period. Neither group returned to baseline ratings identified in Rating 1. When comparing the interaction between the groups at the different time points, there was no strong evidence to suggest a difference in the direction of changed scores (p = 0.431).

**Table 3.** Comparison of ratings of self-perceived performance between immersive and traditional simulation (analysis of variance).

		Rating 1		Rating 2		Rating 3	
	n	Xİ	SD	Xİ	SD	Xİ	SD
**Immersive simulation**	11	11.81	2.56	15.45	1.69	14.09	1.45
**Traditional simulation**	9	13.22	1.79	15.67	2.73	16.00	2.00
**Differences in means (P vs T)**		−1.40		−0.21		−1.91	
**Difference between ratings (p <0.05)**		0.18		0.83		0.02*	

n = number of participants in group; **Xİ** = mean; p = probability; SD = standard deviation; P = 360-degree projection; T = traditional; ^*^ = statistically significant; Rating 1 = before three-day intensive simulation programme; Rating 2 = after three-day intensive simulation programme; Rating 3 = after nine-week period.

While the statistical significance indicates a notable divergence in measures of differences, the practical significance of these findings remains uncertain and requires further investigation, especially for the findings at Rating 3.

## Discussion

This study is the first to directly compare 360-degree projection simulation with traditional methods in the context of paramedicine. The significance of this research lies in its ability to establish baseline measures of self-perceived performance, while also providing a direct comparison to estimate any magnitude of difference between the two methods. Despite the study being considerably underpowered statistically, these findings will be useful for generating hypotheses and for informing the development and design of larger, appropriately powered, prospective studies.

### Differences in self-perceived performance

The results of this study found that second-year paramedicine students rated their overall levels of self-perceived performance higher in the traditional simulation group. This is similar to the findings of Williams et al. (2022), who reported that for the outcome of situational awareness, paramedicine students who participated in traditional methods of simulation reported higher levels than the students who had completed 360-degree projection methods of simulation.

Due to the complexity of the learning experience associated with simulation, further research is needed to determine the reasons for this outcome. However, it could be associated with variations in cognitive load, specifically an increased cognitive demand experienced by participants in the 360-degree projection simulation group. Previous research has indicated that increased cognitive load may affect student performance in simulation (Alconero-Camarero, et al., 2021; Little et al., 2021; Mills et al., 2016). The stimuli used in the traditional setting were not as complex as the additional stimuli provided using 360-degree projection simulation. The increased stimuli associated with the 360-degree projection simulation space could have altered the students’ perception of their performance due to emotional and psychological responses, such as an increase in cognitive load. This may have resulted in a failure to convert stored information from students’ long-term memory into working memory, causing perceptions of increased errors and decreased performance, which translated to their ratings (Fraser et al., 2015).

Yet, the exposure of participants to the increased cognitive demand may be beneficial to student learning in the long term, acting as a stimulus for students to improve their performance. By encouraging greater distractions, increased mental demand and more pressurised environments, students may be able to build enhanced decision-making and resilience strategies before entering the workforce. As a form of extended reality that combines artificial environments alongside physical, tactile elements, this may encourage a replicable stress experience to real-world environments, to prepare students to successfully perform as paramedics in the real world (Leblanc et al., 2012). This would address concerns previously reported by graduates who felt inexperienced before commencing employment as paramedics due to limited exposure (Kennedy et al., 2015).

### The implementation of 360-degree projection simulation into educational design

While the results showed that the traditional simulation group consistently rated themselves higher than the immersive simulation group, further analysis of the interaction between the two groups revealed no strong evidence to suggest that the groups performed differently over time. This indicates that, while a small difference may exist in the perception of students regarding their performance, both forms of simulation provide comparable improvement over time.

Further analysis of the results indicates that the ‘dosage’ of simulation may be more critical than the specific type of simulation employed. This is evidenced in the fact that self-perceived performance did not return to baseline levels even after the nine-week washout period. This finding suggests that the intensity or frequency of the simulation plays a more significant role than the method itself in maintaining performance and achieving long-term observable effects (Sullivan et al., 2019). While there are limited studies that indicate a specific number of simulations to maintain skills over time (Svellingen et al., 2021), previous research has indicated that 18 simulations with hands-on experience and an additional nine in observer roles will result in an increase in perceived confidence and competence after a nine-week repeated simulation intervention (Mould et al., 2011). Therefore, the proposed dose of 20 simulations used in our research may be considered a replicable dose in future interventional studies in both the undergraduate setting and for clinicians who engage in simulation as a form of continuing professional development.

The results also suggest that, in addition to simulation frequency, maintaining a consistent learning environment may be essential for sustaining effective practice. This is shown by the variation in self-perceived performance ratings between groups after the nine-week washout period (Rating 3). Students in the traditional simulation group were consistently exposed to the same type of simulation, known as repeated exposure, in comparison to the 360-degree projection simulation group, who were exposed to both environments during the intensive programme and the nine-week washout period. We hypothesise that individuals in the 360-degree projection group may have not received enough repeated exposure to demonstrate improvement over a prolonged period. Such a result implies that consistency of the environment may be just as important as the simulation design itself and needs to be aligned with the learning needs of the participants. Using a scaffolded approach in simulation design would ensure students feel supported in their development (Erlam et al., 2017). This indicates that the use of 360-degree projection simulation as an adjunct to traditional methods is ideal for final-year students, once they have acquired the necessary knowledge to be translated to a real-world setting.

### The importance of future research

To date, this remains the only study offering a direct comparison between 360-degree projection simulation and traditional simulation. While this research provides interesting insight into the use of 360-degree projection in educational practice, potentially as an adjunct to traditional methods currently used, there is still insufficient evidence to support its design and implementation on a larger scale. At present, the benefit of 360-degree projection simulation spaces is not clear, with limited research regarding the benefits for students and educators and for implementation at an institutional level. Therefore, acknowledging the limitations of this research and the current understanding of technology-enhanced simulation, further collaboration and research are needed to identify best practice for undergraduate and professional education and training.

### Limitations

Although the results showed differences based on statistical analysis, these should be interpreted as exploratory and as a guide to future research. Given the small sample size of the study, caution must be taken when concluding the practical significance in real-world contexts. The limited number of participants, due to the COVID-19 pandemic and public health restrictions that impacted data collection, increases the potential for Type I error. Nonetheless, this study supports future research with larger sample sizes to confirm and extend the current findings and facilitate their applications to real-world settings.

Typical of a pilot study, this research was completed at a single site, which can affect generalisability of findings to the greater population. The students also did not have equal exposure to 360-degree projection simulation and traditional simulation, which should be taken into consideration for future studies. Any future research would benefit from multi-site recruitment, with inclusion of both undergraduate and health professional populations to further broaden the application in the real-world setting. Further, a mixed-method approach to provide context to the experiences of the participants would provide additional insight into their perceptions of the simulation spaces, their overall performance and the factors that contributed to their ratings. Further comparison to objective outcome measures may be considered, in view of the sparsity of evidence that exists in the literature. This would provide a richer analysis of the use of 360-degree projection simulation methods to contribute to an understanding of feasibility and sustainability.

## Conclusion

This pilot study has provided exploratory evidence regarding 360-degree projection simulation and traditional forms of simulation used in the paramedicine context. While this research provides some insight into the use of 360-degree projection simulation, there is still insufficient evidence to support its design and implementation on a larger scale. Given the rapidity of technological development in this area, it is crucial to maintain research momentum in this field to ensure that undergraduate students and other healthcare professionals are given the tools they need to succeed in practice and to safeguard the well-being of the communities they serve.

## Acknowledgements

I would like to thank the participants and the facilitators of this study, who volunteered their time to progress this research. I would also like to thank Dr Paul Fahey, who assisted with the analysis for this research.

## Author contributions

RV was involved in the conception of the idea, the development of the methods and analysis plan, the collection of the data, the analysis of the data and the write-up of the article. PS was involved in the conception of the idea, the development of the methods and analysis plan, the collection of the data, the analysis of the data and the write-up of the article. LT was involved in the conception of the idea, the development of the methods and analysis plan, the collection of the data and the write-up of the article. RV acts as the guarantor for this article.

## Ethics

Ethics approval was received from the University Human Research Ethics Committee (Ref: H14552) before recruiting participants for this research study.

## Funding

This research received funding from the Australasian College of Paramedicine; however, it did not have any involvement with the data collection or analysis associated with this research.
